# Alterations of 5-hydroxymethylation in circulating cell-free DNA reflect molecular distinctions of subtypes of non-Hodgkin lymphoma

**DOI:** 10.1038/s41525-021-00179-8

**Published:** 2021-02-11

**Authors:** Brian C.-H. Chiu, Chang Chen, Qiancheng You, Rudyard Chiu, Girish Venkataraman, Chang Zeng, Zhou Zhang, Xiaolong Cui, Sonali M. Smith, Chuan He, Wei Zhang

**Affiliations:** 1 Department of Public Health Sciences, The University of Chicago, Chicago, IL USA; 2 Department of Preventive Medicine, Northwestern University Feinberg School of Medicine, Chicago, IL USA; 3 Department of Chemistry, The University of Chicago, Chicago, IL USA; 4 Department of Pathology, The University of Chicago, Chicago, IL USA; 5 Section of Hematology/Oncology, Department of Medicine, The University of Chicago, Chicago, IL USA; 6 Department of Biochemistry and Molecular Biology; Institute for Biophysical Dynamics; and Howard Hughes Medical Institute, The University of Chicago, Chicago, IL USA

**Keywords:** Epigenomics, Epidemiology, B-cell lymphoma

## Abstract

The 5-methylcytosines (5mC) have been implicated in the pathogenesis of diffuse large B-cell lymphoma (DLBCL) and follicular lymphoma (FL). However, the role of 5-hydroxymethylcytosines (5hmC) that are generated from 5mC through active demethylation, in lymphomagenesis is unknown. We profiled genome-wide 5hmC in circulating cell-free DNA (cfDNA) from 73 newly diagnosed patients with DLBCL and FL. We identified 294 differentially modified genes between DLBCL and FL. The differential 5hmC in the DLBCL/FL-differentiating genes co-localized with enhancer marks H3K4me1 and H3K27ac. A four-gene panel (*CNN2, HMG20B, ACRBP*, *IZUMO1*) robustly represented the overall 5hmC modification pattern that distinguished FL from DLBCL with an area under curve of 88.5% in the testing set. The median 5hmC modification levels in signature genes showed potential for separating patients for risk of all-cause mortality. This study provides evidence that genome-wide 5hmC profiles in cfDNA differ between DLBCL and FL and could be exploited as a non-invasive approach.

## Introduction

Non-Hodgkin Lymphoma (NHL) is a heterogeneous group of malignancies that arises within lymphoid cells in the bone marrow or more mature cells in the peripheral lymphoid organs. The two most common types of NHL are diffuse large B-cell lymphoma (DLBCL) and follicular lymphoma (FL), accounting for ~35% and ~20% of all NHLs, respectively^1^. Although patterns of occurrences and intensive epidemiology research suggest etiologic commonality and heterogeneity for DLBCL and FL^2,3^, risk factors for these two subtypes remain incompletely understood. Evaluating the molecular differences between DLBCL and FL offers opportunities to improve our understanding of pathogenesis of these two major types of NHL.

Epigenetic modifications, particularly the methylation of cytosines in DNA, i.e., 5-methylcytosines (5mC), have been implicated in the pathobiology of NHL^4^. Most epigenetic studies on NHL, however, have focused on 5mC or have interpreted all modified cytosines as 5mC due largely to the lack of enabling technologies that can robustly distinguish 5mC from other modified cytosines, particularly 5-hydroxymethylcytosines (5hmC), a class of modified cytosines that are generated from 5mC in an active demethylation process through the ten-eleven translocation (TET) enzymes^5^. Unlike 5mC which is recognized for its role in repressing not only protein-coding genes but also a vast amount of transposons in the human genome^6^, 5hmC modifications are associated with active gene regulation^7^. A recent study of a 5hmC map of human tissues showed that 5hmC is enriched preferentially in tissue-specific gene bodies and enhancers^8^, representing a distinct genome-wide distribution from 5mC. Global and focal differential 5hmC modifications have been observed in several cancers, including hematological malignancies^9^. Higher levels of 5hmC prohibit cell division by maintaining cells in G1 for a longer period^10^, thus directly contributing to the balance between cell proliferation and apoptosis. Recent studies on 5hmC also revealed the complexes that form between transcription factors and epigenetic regulators during B-cell differentiation^11,12^, suggesting a potential role of 5hmC in B-cell malignancies. Therefore, profiling genome-wide 5hmC could provide insights on direct epigenetic landscape and adds additional value to the epigenetic modifications between DLBCL and FL. However, due partly to technical constraints (e.g., bisulfite conversion based Illumina microarray), the majority of epigenetic studies of NHL do not distinguish 5hmC from 5mC or interpreted all modified cytosines as 5mC.

Studies^13–17^ have demonstrated that the 5hmC-Seal technique combined with next generation sequencing is a sensitive and robust technique for genome-wide 5hmC distributions in clinical specimens from different sources, such as circulating cell-free DNA (cfDNA) in peripheral blood plasma or genomic DNA from tissue biopsies. Using 5hmC-Seal, we profiled 5hmC profiles in cfDNA from plasma and demonstrated that 5hmC profiles at the time of diagnosis are associated with all-cause mortality in patients with DLBCL^18^, supporting the utility of this technique for investigating 5hmC and cfDNA as a promising source for molecular analysis of tumor epigenetic alterations^13^.

In the current work, we profiled genome-wide 5hmC in cfDNA derived from plasma from 73 newly diagnosed patients with de novo DLBCL and FL. We analyzed genomic features to identify differential 5hmC modifications between DLBCL and FL. We also investigated canonical pathways^19^, Gene Ontology^20^ biological processes, and functional interaction networks for the differentially modified genes between DLBCL and FL. The identified subtype-differentiating signatures were evaluated for their co-localization with *cis*-regulatory elements to inform about biological connections between 5hmC and gene regulatory machinery. We also explored the detected subtype-differentiating genes for their prognostic value to evaluate their potential clinical implications in NHL.

## Results

### Characteristics of the study subjects

There were no significant differences between DLBCL (*n* = 48) and FL (*n* = 25) with respect to gender distribution, race/ethnicity, and stage (Table 1). The median age at the time of diagnosis for patients with FL (51.0 years) was lower than that of DLBCL (59.0 years). Among the 48 patients with DLBCL, 34 had cell-of-origin determined based on the Han’s algorithm^21^. Of those, 23 were germinal center B-cell-like (GCB) DLBCL and 11 patients were activated B-cell-like (ABC) DLBCL. In addition, 28 (38.4%) had an elevated LDH levels (cut-off: ≥245 U/L) at the time of diagnosis.

**Table 1:** Demographics and clinical characteristics of the study subjects.

Characteristics	DLBCL, *n* = 48 (%)	FL, *n* = 25 (%)	*P*-value
Age (yr)			0.12
Mean (SD)	58.1 (±14.1)	53.2 (±12.8)	
Median (min, max)	59.0 (24, 82)	51.0 (32, 76)	
Sex			1.00
Males	30 (62.5)	15 (60.0)	
Females	18 (37.5)	10 (40.0)	
Race			0.83
EA	39 (81.3)	19 (76.0)	
Non-EA	9 (18.8)	6 (24.0)	
Cell of origin			–
GCB	23 (47.9)	–	
ABC	11 (22.9)	–	
Missing	14 (29.2)	–	
Stage			0.10
I & II	17 (35.4)	5 (20.0)	
III & IV	20 (41.7)	17 (68.0)	
Missing	11 (22.9)	3 (12.0)	
LDH			0.004
Elevated (≥245 U/L)	25 (52.1)	3 (12.0)	
Not elevated (<245 U/L)	22 (45.8)	21 (84.0)	
Missing	1 (2.1)	1 (4.0)	
Vital status			0.18
Alive	34 (70.8)	23 (92.0)	
Dead	12 (25.0)	2 (8.0)	
Missing	2 (4.2)	–	

*P*-value of age is based on Student’s *t*-test. *P*-values of sex, race, stage, and LDH are based on the Chi-square test.

*DLBCL* diffuse large B-cell lymphoma, *FL* follicular lymphoma, *EA* European American, *LDH* lactic acid dehydrogenase.

### Differential 5hmC between DLBCL and FL

**Fig. 1:**
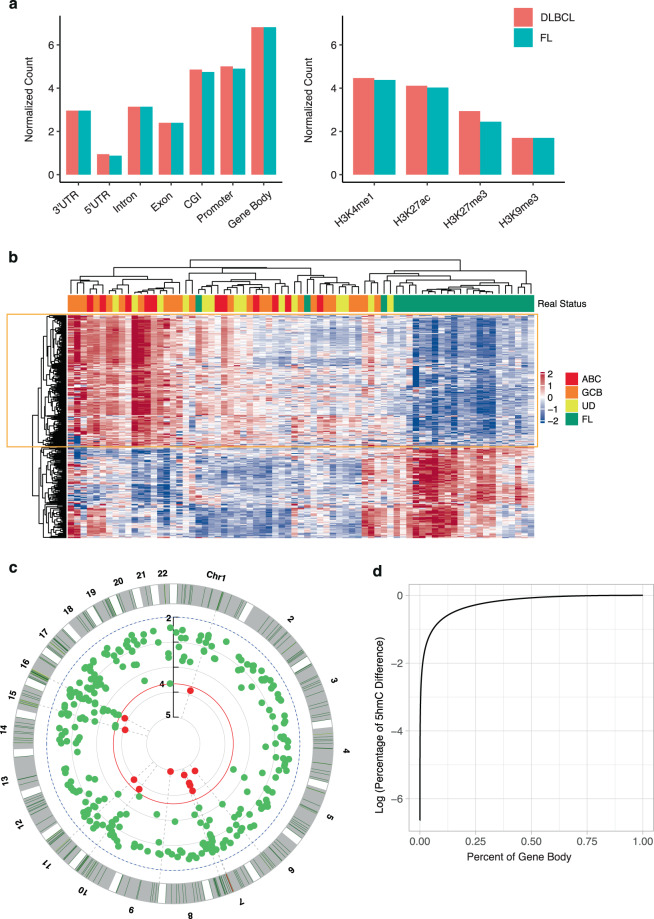
Overview of 5hmC in cfDNA between DLBCL and FL. **a** Normalized counts of 5hmC reads show distinct distributions in the human genome. **b** The 294 differentially modified genes between DLBCL and FL are shown. **c** The distribution of the 294 differentially modified genes between DLBCL and FL is shown across the human genome. **d** A small proportion of genes drive the overall differential modification between DLBCL and FL. Red indicates differentially modified genes with *P*-value < 1 × 10^−4^. 3′TR 3′ untranslated region, 5′UTR 5′ untranslated region, CGI CpG islands, DLBCL diffuse large B-cell lymphoma, FL follicular lymphoma, UD DLBCL of unknown cell-of-origin, GCB germinal center B-cell-like DLBCL, ABC activated B-cell-like DLBCL.

The 5hmC-seal sequencing reads in cfDNA from plasma for patients with DLBCL and FL showed a distinct genomic distribution, displaying more normalized counts located in gene bodies relative to other components of genic regions, such as UTRs, promoters, and introns (Fig. 1a). Based on the B-cell data from the Roadmap Epigenomics Project, we found that 5hmC tended to accumulate in histone modifications that mark active expression (e.g., H3K4me1 and H3K27ac), relative to repressive markers (e.g., H3K9me3 and H3K27me3) (Fig. 1a), an observation that is consistent with previous reports^14,15,18^. Differential analysis of the 17,698 gene bodies with non-zero variance found 294 genes showing differential modification between DLBCL and FL (Fig. 1b) at the permutation-based empirical *P*-value < 0.05 and fold-change >20% (Fig. 1c, Supplementary Table 1). Among these 294 differentially modified genes, we found that the cytobands 15q13.2 (6 genes), 7q11.23 (9 genes), 15q13.3 (5 genes), and 16p11.2 (8 genes) were enriched (FDR < 5%) relative to the reference genome (Supplementary Table 2). Overall, 16,835 of 17,698 genes showed a trend of decreasing modification level of 5hmC in FL than DLBCL, and 50% of the total 5hmC differential modification between DLBCL and FL was driven by a small proportion of genes (~10%) (Fig. 1d).

**Fig. 2:**
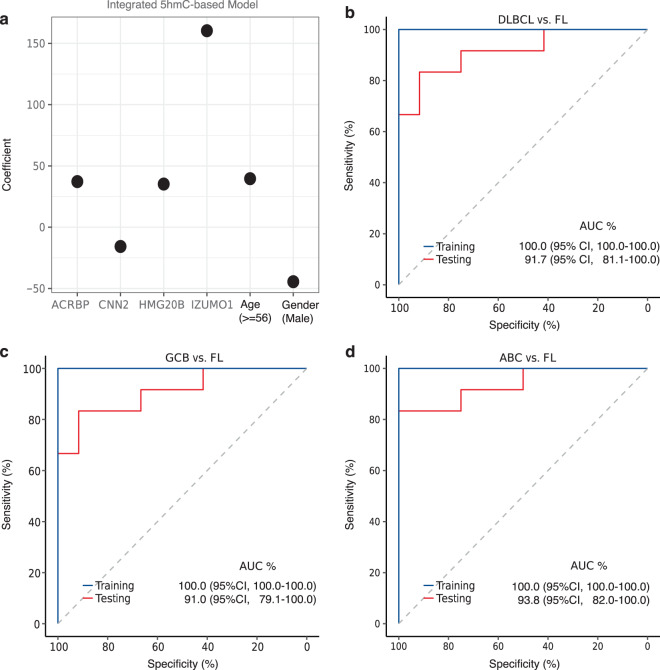
An integrated 5hmC-based model summarizes differential epigenetics in cfDNA between DLBCL and FL. **a** The covariates and coefficients of the integrated model are shown. The performance of the integrated 5hmC-based model is shown for distinguishing: **b** DLBCL vs. FL; **c** GCB-type DLBCL vs. FL; and **d** ABC-type DLBCL vs. FL. DLBCL diffuse large B-cell lymphoma, FL follicular lymphoma, GCB germinal center B-cell-like DLBCL, ABC activated B-cell-like DLBCL, AUC area under curve, CI confidence interval.

Next, to summarize the differentially modified 5hmC profiles into an integrated signature, we performed further feature selection from the 294 differential genes between DLBCL and FL. We randomly divided the samples into two sets: a training set of 37 samples (DLBCL, *n* = 24; FL, *n* = 13) and a testing set of 36 samples (DLBCL, *n* = 24; FL, *n* = 12), with balanced distributions of age and gender between the two sets. A weighted model (Fig. 2a) comprising age, gender, and four signature genes, including *ACRBP* (encoding acrosin binding protein), *IZUMO1* (encoding Izumo sperm-egg fusion protein 1), *CNN2* (encoding calponin 2), and *HMG20B* (encoding high mobility group 20B), was constructed from the training set to represent the overall 5hmC differential modification pattern between DLBCL and FL. This integrated model showed an 100% AUC in the training set and an AUC of 91.7% (95% CI, 81.1–100.0%) in the testing set in differentiating DLBCL from FL (Fig. 2b). This four-gene model also achieved similar performance in distinguishing GCB-type DLBCL from FL and ABC-type DLBCL from FL (Fig. 2c, d). In a subset of patients (59 out of 73) with available information on stage, this four-gene model showed comparable performance distinguishing late stage (stage 3/4) DLBCL from FL with an AUC of 95.1% (95% CI, 86.0–100.0%) and early stage (stage 1/2) DLBCL from FL with an AUC of 84.0% (95% CI, 65.4–100.0%) in the testing set (Supplementary Fig. 1).

### Linking differential signatures with local regulatory elements

**Fig. 3:**
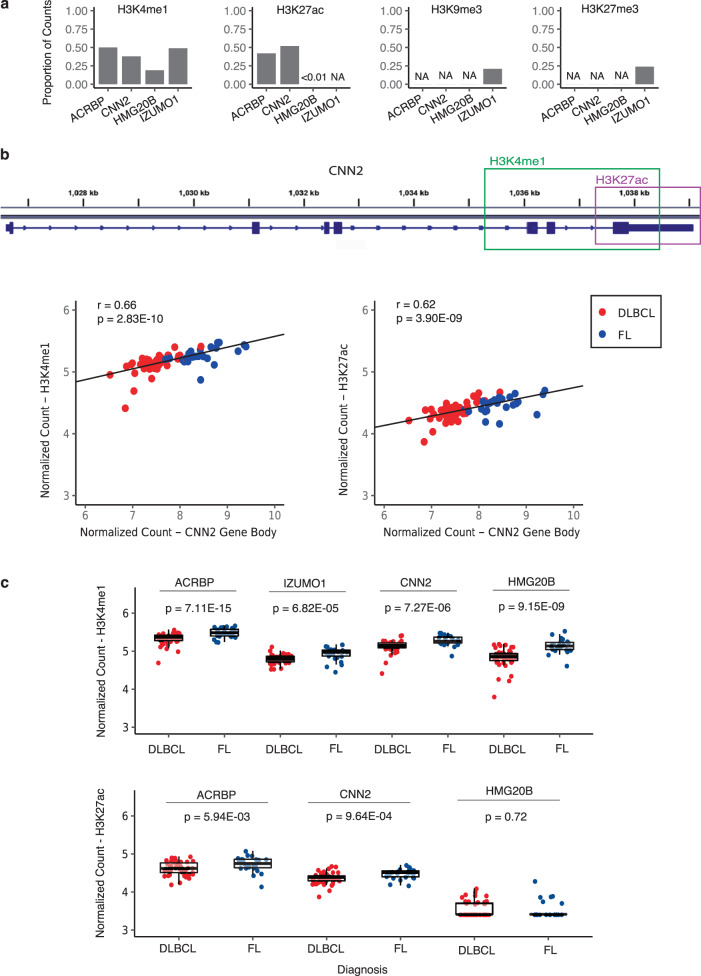
Gene regulatory relevance of the differentially modified 5hmC in signature genes. **a** The proportion of 5hmC read counts in a particular histone modification mark relative to the read counts in the gene body is shown for each signature gene. **b** The 5hmC read counts in *CNN2* are co-localized with enhancer markers (e.g., H3K4me1 and H3K27ac). **c** The 5hmC read counts co-localized in respective *cis*-regulatory elements show differential modification between DLBCL and FL. The coordinates of histone modification marks are based on the narrow peaks from the Roadmap Epigenomics Project’s B-cell data (accessed on July 25, 2020). Genomic positions are based on the human genome reference (hg19). Statistical significance of the difference between DLBCL and FL was tested using the Wilcoxon signed-rank tests. DLBCL diffuse large B-cell lymphoma, FL follicular lymphoma.

Several *cis*-regulatory elements, including H3K4me1, H3K27ac, H3K27me3, and H3K9me3, were summarized for 5hmC read counts in gene bodies of the four signature genes that differentiate DLBCL and FL (Fig. 3a). Overall, the 5hmC reads within these four signature genes showed a significant trend of co-localizing with histone modifications that mark enhancers and active expression (i.e., H3K4me1and H3K27ac), compared to repression markers (i.e., H3K27me3 and H3K9me3) (Fig. 3a). The 5hmC reads in the gene bodies of the four signature genes were primarily co-localized with H3K4me1 (36.9%) and H3K27ac (35.9%), and highly correlated with the read counts in enhancer markers (Supplementary Table 3). Using *CNN2* as an example, the peaks of H3K4me1 and H3K27ac were located near the 3′-end, with an overlapping region of ~1 kb. The distribution of 5hmC in the gene body of *CNN2* showed a strong correlation with H3K4me1 (Pearson’s *r* = 0.66, *P* < 0.001) and H3K27ac (Pearson’s *r* = 0.62, *P* < 0.001), indicating a link between 5hmC and the local regulatory elements (Fig. 3b).

Furthermore, the 5hmC reads co-localized with H3K4me1 and H3K27ac within the signature genes also showed a trend of differential modification between DLBCL and FL (Fig. 3c). For example, the normalized counts of the 5hmC reads co-localized with both H3K4me1 and H3K27ac in *CNN2* were significantly higher in FL than DLBCL (*P*-value < 0.001), consistent with the direction of differential modification in the gene body. Similar patterns were observed in other signature genes, with the exception of *HMG20B* which showed no difference in 5hmC co-localized with H3K27ac between DLBCL and FL, reflecting that there were few 5hmC reads located in H3K27ac within *HMG20B*.

### Functional exploration of the differential 5hmC between DLBCL and FL

**Fig. 4:**
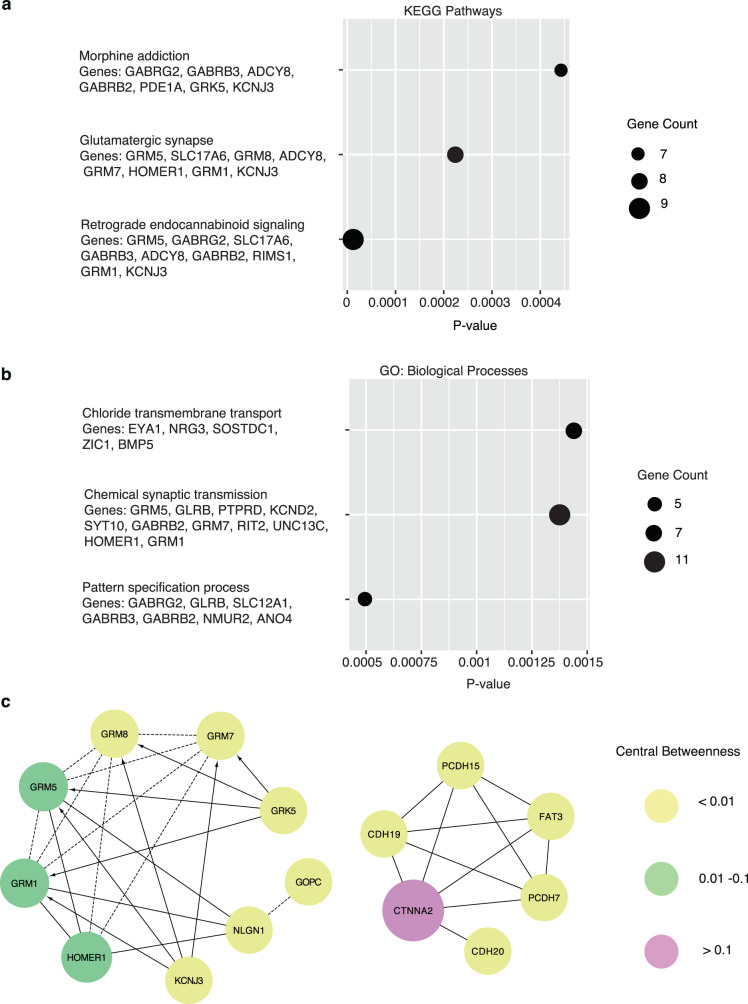
Functional relevance of the differentially modified genes between DLBCL and FL. Enriched pathways and biological processes are observed among the 294 differentially modified genes between DLBCL and FL: **a** KEGG pathways; and **b** GO biological processes. **c** Reactome FI network analysis detects links among a subset of differentially modified genes. The node size is proportional to the central betweenness measurement that denotes the importance of a gene in the FI network. KEGG Kyoto Encyclopedia of Genes and Genomes, GO gene ontology, FI functional interaction, DLBCL diffuse large B-cell lymphoma, FL follicular lymphoma.

We conducted functional annotation analysis of the 294 differential genes using the NIH/DAVID tool and identified enrichment in certain canonical pathways from the KEGG, including “retrograde endocannabinoid signaling”, “morphine addiction”, and “glutamatergic synapse” (Fig. 4a), as well as GO biological processes, including “chloride transmembrane transport”, “chemical synaptic transmission”, and “pattern specification process” (Fig. 4b), which were enriched among the 173 genes with higher 5hmC modification levels in DLBCL than FL. In contrast, the 121 genes with higher 5hmC modification levels in FL than DLBCL were enriched in the biological process of “cellular protein metabolic process” (Supplementary Table 4). Moreover, results from the Reactome FI network analysis indicated that several functional hubs played important roles among the 294 differentially modified genes. For example, *CTNNA2* (encoding catenin alpha 2) was found to be a hub gene of the *CDH* (encoding cadherin) and *PCDH* (encoding procadherin) families, while *HOMER1* is a hub gene of the differentially modified genes such as *NLGN1* (encoding neuroligin 1) and the GRM (encoding glutamate metabotropic receptor) family (e.g., *GRM1*, *GRM5*, *GRM7*, and *GRM8*) (Fig. 4c). Notably, the indicated Reactome FI networks were consistent with the NIH/DAVID results, such as the enrichment of “glutamatergic synapse”.

### Clinical implications of the differential 5hmC between DLBCL and FL

We explored whether these four marker genes that differentiate DLBCL from FL also have clinical implications by comparing overall survival (OS) between subjects with higher vs. lower than median modification level (i.e., normalized 5hmC-Seal counts) of each marker gene. Although none of 5hmC levels in these signature genes were statistically associated with OS, Kaplan–Meier survival curves showed potential of discriminating patients for all-cause mortality based on the median 5hmC modification level (Supplementary Fig. 2a–d). Of note, the majority of FL patients were assigned to the low-risk group based on the 5hmC levels of signature gene alone. The credence of these findings is supported by those from survival curves of clinical indices that older age, males, and later stage were associated with worse OS (Supplementary Fig. 2e–g).

## Discussion

We investigated epigenetic alterations underlying the molecular distinctions between patients with DLBCL and FL using a state-of-the-art 5hmC-profiling technique in patient-derived cfDNA. There is evidence that 5hmC could be differentially regulated in hematological malignancies^22,23^, suggesting an unbiased investigation of genome-wide 5hmC could provide insights into the molecular characteristics of DLBCL and FL. We found that 294 genes were differentially modified between DLBCL and FL. Of these, a four-gene panel (*CNN2, HMG20B, ACRBP*, *IZUMO1*) robustly represented the overall 5hmC modification pattern that distinguishes DLBCL and FL.

Our genome-wide scan of 5hmC modifications between DLBCL and FL identified 294 genes that showed differential modification levels in their gene bodies. These 294 differentially modified genes were not uniformly distributed across the human genome, instead they appeared to be enriched in several specific cytobands, especially the 11 genes on 15q13.2-3 that features several genes from the Golgin A8 family. As an organelle that plays a central role in synthesis and secretion of macromolecules, the Golgi complex regulates membrane trafficking and cellular sorting by interacting with death receptor families of transmembrane proteins such as CD95, thus regulating the expression of CD95 in B lymphocytes^24^. Because loss of CD95 expression/function has been associated with a more aggressive tumor grade^25^, the enrichment of Golgi complex-related genes suggests that they may contribute to the molecular distinctions between DLBCL and FL through genes involving cellular adhesion/junction and signaling receptors on membrane. The Atlas Genetics and Cytogenetics in Oncology and Haematology database also reported the linkage of 15q13 with DLBCL and FL^26^. Our findings provide additional evidence of 5hmC in these genes that might contribute to lymphomagenesis.

We find that several canonical pathways and GO biological processes were enriched among the 294 differentially modified genes, including “retrograde endocannabinoid signaling”, “morphine addiction”, and “glutamatergic synapse”, the last of which was also an enriched FI network from the Reactome database. For example, *ADCY8* (encoding adenylate cyclase 8), a gene involved in multiple pathways, is one of the calcium-sensitive protein isoforms that regulates the phosphorylation of CREB^27^, which plays an critical role in lymphoma by binding to the promoter of translocated bcl-2 but not normal alleles in FL and transformed lymphomas^28^. Importantly, the enrichment of genes involving metabolism, specifically glutamate metabolism, suggests a link between 5mC oxidation and 5hmC formation and the mechanism of metabolic reprogramming in NHL pathogenesis^29^. Moreover, the enrichment of genes involving retrograde endocannabinoid signaling revealed the potential relevance of 2-arachidonoylglycerol, an endocannabinoid, in NHL, particularly in DLBCL through epigenetic alterations^30^.

Our analysis demonstrated that an integrated model comprised of multiple 5hmC marker genes could summarize the genome-wide 5hmC distinctions between DLBCL and FL. Specifically, a four gene-based model showed an excellent distinguishing capacity for DLBCL and FL, regardless of the cell-of-origin or stage of DLBCL. These four signature genes were also functionally relevant to NHL. For example, *CNN2* is a regulator for actin cytoskeleton that is involved in morphological changes during the lymphoblastic transformation of FL^31^, while gene expression profiling of DLBCL indicates that the HMG family plays a role in germinal center development of B-cell^32^. These four signature genes may also have prognostic value because Kaplan–Meier survival curves showed potential for separating patients for all-cause mortality using the median modification levels of the signature genes alone. Interestingly, all FL patients were grouped in the low-risk group of all-cause mortality based on the 5hmC levels of *CNN2* and *HMG20B*. For *CNN2*, of the 10 DLBCL patients in the low-risk group of death, 4 of the 5 patients with known cell-of-origin data were the GCB-type, which typically has better prognosis than the ABC-type of DLBCL. However, these findings should be interpreted cautiously because due to small sample size, we were not able to conduct stratification analysis by DLBCL and FL, nor can we conduct survival analysis controlling for prognostic factors (e.g., International Prognostic Index, cell-of-origin, etc.).

Results of *cis*-regulatory elements indicated a potential mechanism underlying the differential modification of 5hmC and transcriptional relevance of these epigenetic modifications. Specifically, the histone modifications that mark enhancers and active expression appeared to be co-localized with the 5hmC reads profiled in the differential genes, and contributed to the alterations between DLBCL and FL. Although the current analysis focused on 5hmC, our findings suggest a potential link between 5hmC formation and dysregulation of target genes through altered epigenetic status of *cis*-regulatory elements.

To the best of our knowledge, no study has evaluated genome-wide 5hmC distinctions between DLBCL and FL using patient-derived cfDNA. Our findings of a four-gene panel that distinguished DLBCL and FL warrant validation in larger patient populations. For example, limited sample size prohibited the current report to validate gene signatures using independent samples. Similarly, our findings of potential for separating patients for all-cause mortality using the median modification levels of the signature genes alone should be interpreted cautiously. Another limitation due to relatively small sample size is that we were not able to investigate DLBCL patients transformed from FL which would likely provide new insights into the epigenetic mechanism of lymphomagenesis because transformed DLBCL has a distinct gene expression profile from de novo DLBCL^33^. Finally, although cfDNA represents a clinically convenient methodology for investigating epigenetic modifications, the lack of simultaneous profiling of gene transcription limited further exploration of the molecular mechanisms that distinguish DLBCL from FL.

In conclusion, the current work underscores the substantial contribution of 5hmC, an understudied epigenetic modification, to the molecular distinctions between DLBCL and FL. Our findings not only enhanced our understanding of the molecular differences between DLBCL and FL through epigenetic modification, but also demonstrated a potential for utilizing the 5hmC-Seal, a highly sensitive technique that requires limited material (e.g., a few nanograms of cfDNA from <5 mL of plasma) as a non-invasive clinical approach in the precision medicine of NHL.

## Methods

### Study subjects

We prospectively enrolled adult patients ≥20 years old who were newly diagnosed with NHL at the University of Chicago Medical Center (UCMC) from 2010 to 2013. All diagnoses were confirmed by hematopathologists according to the 2008 World Health Organization criteria^34^. Informed consent was obtained from all participants. Blood samples were drawn from consented patients and processed immediately to separate plasma. The current report included 73 patients (de novo DLBCL, *n* = 48; FL, *n* = 25) with plasma available for cfDNA extraction (Table 1). Patients with primary central nervous system lymphoma, post-transplant lymphoproliferative disorder, transformation of a previously diagnosed indolent lymphoma, or HIV infection were excluded. Data on clinical and pathologic characteristics of subjects at the time of diagnosis, such as lactic acid dehydrogenase (LDH) level, Ann Arbor stage^35^, and tumor cell-of-origin were collected from electronic health records. This study was approved by the Institutional Review Board of the University of Chicago.

### Sample preparation, 5hmC-Seal, and sequencing

Details about cfDNA sample preparation, 5hmC-Seal library construction, and subsequent sequencing have been described in our previous publications^14,15^. Briefly, ~2–3 mL of frozen plasma from each subject was processed by centrifuging at 1350 × *g* for 12 min twice and at 13,500 × *g* for 12 min once, followed by cfDNA extraction (~2–4 ng/sample) using the QIAamp Circulating Nucleic Acid Kit (Qiagen, Germany). The 5hmC-Seal libraries were constructed according to an established protocol^14,36^. DNA samples were first repaired and ligated with adaptors. Next, T4 bacteriophage enzyme β-glucosyltransferase was used to transfer an engineered glucose moiety containing an azide-glucose to 5hmC in duplex DNA. A biotin tag was added to the azide group using Huisgen cycloaddition (“Click”) chemistry. Finally, the 5hmC-containing DNA fragments with biotin tags were captured by streptavidin beads. The 5hmC-Seal libraries were constructed through PCR amplification and paired-end sequenced using the Illumina NextSeq 500 platform (PE50) at the University of Chicago Genomics Core Facility. The cfDNA samples were randomly labeled for the 5hmC-Seal library construction and sequencing. Technicians did not have access to clinical outcomes. Technical robustness, including reproducibility and spike-in controls, of the 5hmC-Seal have been demonstrated in our previous studies^14,36,37^.

### 5hmC-Seal data processing

Bioinformatics processing of the 5hmC-Seal data from cfDNA was described in detail in our previous report^14^. Briefly, raw sequencing reads were trimmed for adaptor sequences using Trimmomatic^38^. Low-quality bases were also trimmed to a minimum length of 30 base pairs (bp), followed by alignment to the human genome reference (hg19) by the GENCODE Project^39^ using Bowtie2’s end-to-end alignment mode^40^. Alignments with Mapping Quality Score ≥10 were counted for gene bodies which were our primary targets because they were shown to be a reliable genomic feature for the 5hmC-Seal data^14^ according to the gene start and gene end annotations using featureCounts^41^ without strand information. Other genomic features, such as promoters, untranslated regions (UTRs), and selected histone modifications (H3K4me1, H3K27ac, H3K9me3, and H3K27me3) were also summarized for comparison. The 5hmC-Seal libraries were sequenced to produce a median of ∼25 million reads in each sample with a median number of ∼13.5 million unique reads mapped to ∼22,000 gene body features. The raw count data were then normalized using DESeq2 (v1.22.2) and corrected for library size.

### Identification of 5hmC differentially modified between DLBCL and FL

The normalized 5hmC-Seal count data of 17,698 gene bodies with non-zero variance from the 73 samples were used to identify informative genes with differential modification (*P*-value < 0.005 and fold-change >20%) between FL and DLBCL using DESeq2^42^, adjusting for batch, age, and gender. Empirical *P*-values based on 10,000 permutations were computed to evaluate the significance of differential genes. The Manhattan plot of the top-ranked differentially modified genes was prepared using the CMplot package^43^. To evaluate whether an integrated 5hmC signature represent the differential 5hmC landscape between DLBCL and FL, we performed further feature selection by applying the elastic net regularization to the multivariate logistic regression models (Eq. (1)), with 10-fold cross-validation using the *glmnet* package (v3.0.2)^44^ in the R Statistical Environment (v3.6.1)^45^:

1 $$\log \frac{{{\mathrm{Pr}}({G} = 1|{x})}}{{{\mathrm{Pr}}({\mathrm{G}} = 0|{\mathrm{x}})}} = {\beta}_0 + {x}^{r}{\beta},$$

where *x* is a *J*∈(1…*j*) by *I*∈(1…*i*) matrix of 5hmC level at gene *j* for sample *i*.

The model is solved by

2 $$\begin{array}{*{20}{c}} {\min } \\ {\left( {{\beta }}_0,\;{\beta} \right)} \end{array}\left[\frac{2}{{2{N}}} = \mathop {\sum}\limits_{{\mathrm{i}} = 1}^{N} {(y^i - {\beta}_0 - {\mathrm{x}}_i^{\mathrm{r}}{\beta})^2 + {\lambda}P_{\alpha}({\beta})} \right],$$

3 $${P}_{\alpha}({\beta}) = \mathop {\sum}\limits_{{j} = 1}^{p} {v_j\left[\frac{1}{2}\left( {1 - {\alpha}} \right){\beta}_j^2 + {\alpha}|{\beta}_j|\right]} ,$$

where *P*_*α*_ is the blend of the lasso (*α* = 1) and ridge (*α* = 0) penalty. The parameter *λ* controls the overall strength of penalty (Eq. (2)), while the parameter *α* controls for the relative proportion between the lasso and ridge penalty (Eq. (3)). This procedure was repeated 200 times, and genes selected in at least 90% iterations were retained as final signature genes.

The receiver operating characteristic (ROC) curves and 95% confidence intervals (CI) were computed to evaluate the differentiating capacity of the integrated 5hmC signature^46^.

### Exploring co-localization of 5hmC with *cis*-regulatory elements

To explore the gene regulatory relevance of 5hmC in cfDNA, we summarized the 5hmC-Seal data to various genomic features, such as *cis*-regulatory element H3K4me1 and H3K27ac from the narrow peak coordinates of Roadmap Epigenomics Project B-cell data, by combining nearby peaks (<200 bp)^47^ overlapped with the four signature genes based on the human genome reference (hg19). The summarized read counts for *cis*-regulatory elements were normalized using DESeq2^42^ and output in log_2_ scale. For each of the final signature genes in the integrated 5hmC panel that differentiate DLBCL and FL, the summarized 5hmC profiles in the gene body were individually tested for correlation with the normalized reads localized within various *cis*-regulatory elements using the Pearson’s correlation test. The Integrative Genome Viewer^48^ was used to visualize the distributions of regulatory elements across a genomic region. For each *cis*-regulatory element, the difference between DLBCL and FL was tested by the Wilcoxon signed-rank test.

### Functional relevance of DLBCL/FL-differentiating 5hmC genes

We explored functional relevance of the differentially modified genes between DLBCL and FL to biological pathways and processes using the Database Visualization and Integrated Discovery (DAVID) tool^49^ for the Kyoto Encyclopedia of Genes and Genomes (KEGG) pathways^19^ and Gene Ontology (GO) biological processes^20^. An enriched pathway or GO biological process was defined as having at least five genes at 5% false discovery rate (FDR). The reactome functional interaction (FI) plug-in from Cytoscape^50^ was used to investigate the functional interactions across the differentially modified genes. The measurement of “betweenness centrality”, which quantifies the importance of a particular node in a network, was used to evaluate the importance of gene hubs in a Reactome FI network.

### Exploring clinical implications of DLBCL/FL-differentiating 5hmC genes

We further explored the association between the component genes of the integrated 5hmC signature and outcomes of patients. Deaths were determined using the National Death Index. OS was defined as the time from initial diagnosis until death from any cause. Follow-up was through December 31, 2017. Subjects were grouped using the signature gene’s 5hmC-Seal profile median read counts. The association between a given gene and OS was assessed using the multivariate Cox Proportional Hazards model to calculate hazard ratios (HR) and 95% confidence intervals (CI), controlling for age at the time of diagnosis, gender, and stage. Kaplan–Meier survival plots and log-rank *P*-values were calculated using the *survival* package^51^ in the R Statistical Environment (v3.6.1)^45^. All tests are two-sided.

### Reporting summary

Further information on research design is available in the Nature Research Reporting Summary linked to this article.

## Supplementary information

Supplementary Information

Reporting Summary

## Data Availability

The individual-level raw and processed 5hmC-Seal profiles have been deposited into the NCBI Gene Expression Omnibus database (Accession Number: GSE155228).

## Code availability

The code related to feature selection, classifier detection, and modeling is implemented in the R Statistical Environment (v3.6.1), which is available upon request.
