# Supplementary material for: Alterations of 5-hydroxymethylation in circulating cell-free DNA reflect molecular distinctions of subtypes of non-Hodgkin lymphoma
Source: NPJ Genom Med. 2021 Feb 11;6:11. doi: 10.1038/s41525-021-00179-8 (PMC7878492; doi:10.1038/s41525-021-00179-8)
Supplement: Supplementary file 1 — Supplementary Information [file 41525_2021_179_MOESM1_ESM.pdf]

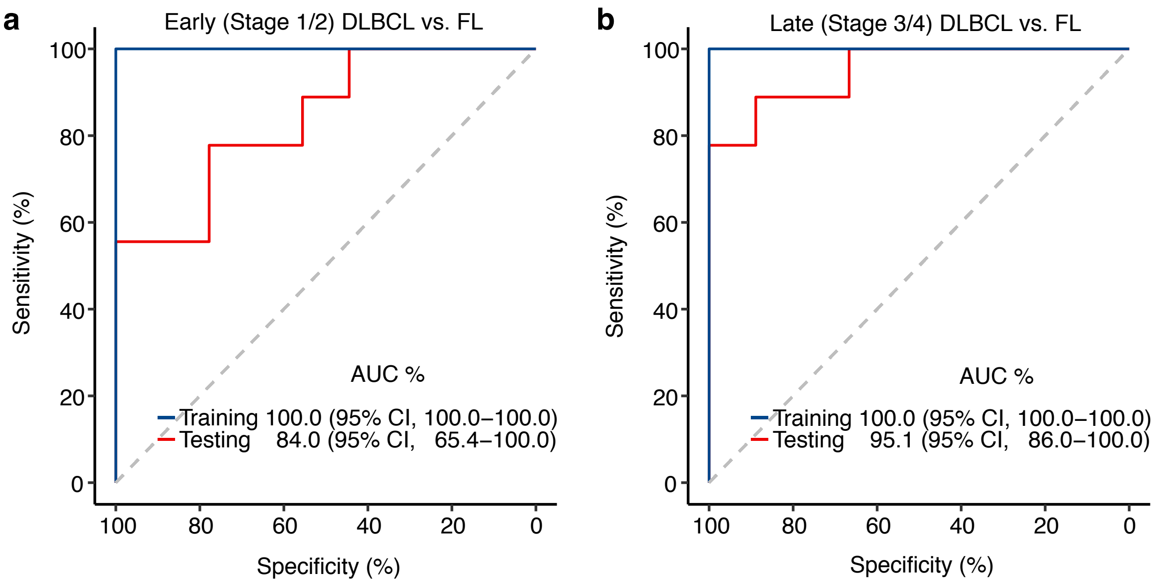

Supplementary Figure 1. An integrated 5hmC-based model distinguished FL from DLBCL by stage. The performance of the integrated 5hmC-based model is shown for distinguishing: (**a**) Early (Stage 1/2) DLBCL vs. FL; and (**b**) Late (Stage 3/4) DLBCL vs. FL. DLBCL: diffuse large B-cell lymphoma; FL: follicular lymphoma; AUC: area under curve; CI: confidence interval.

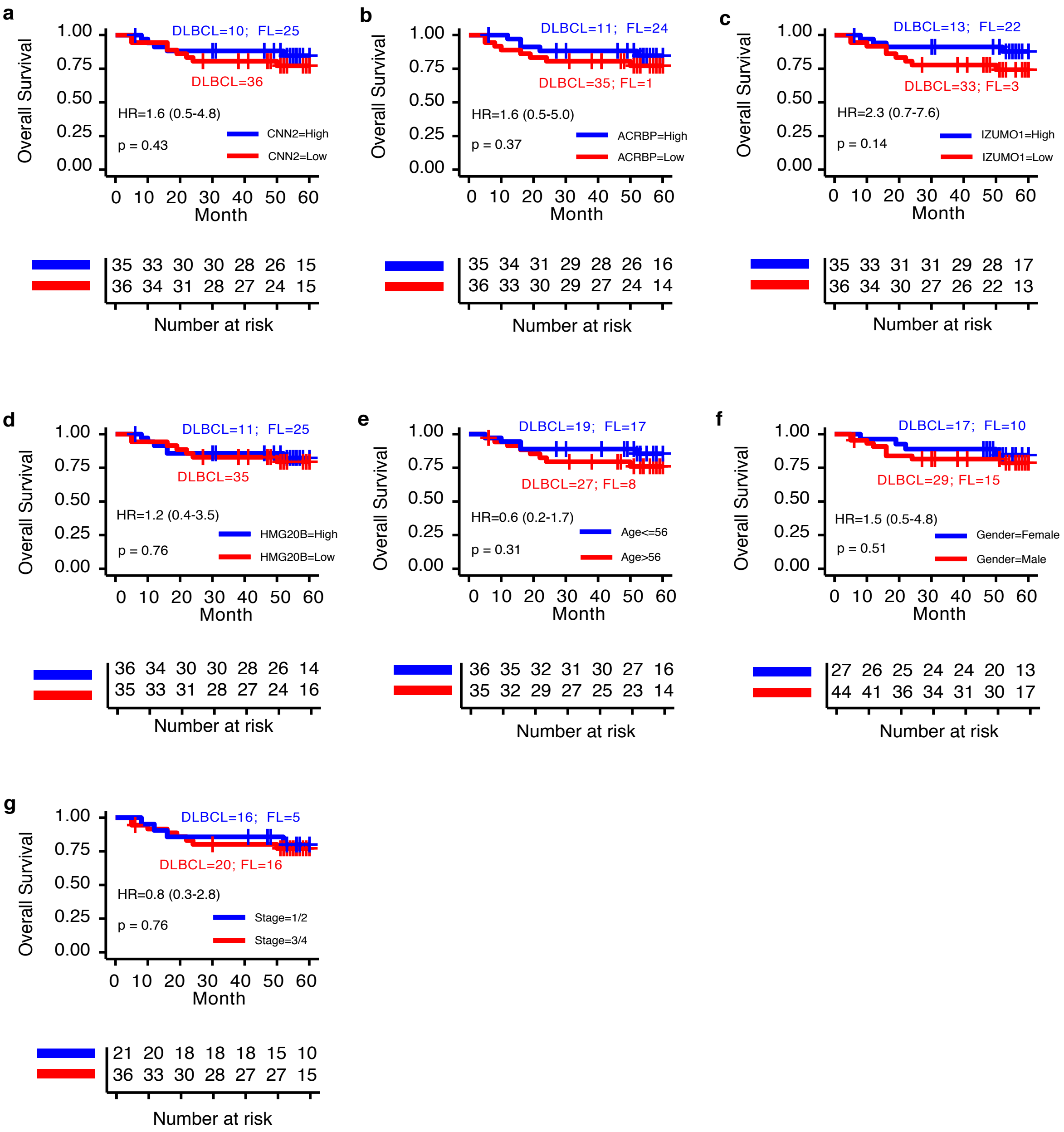

Supplementary Figure 2. Functional exploration of the differential 5hmC between DLBCL and FL. The level of 5hmC modification in each signature gene is explored for association with the overall survival (OS) of patients, grouped by the median of normalized 5hmC-Seal counts. Vital status was available for 71 of the 73 subjects. The low-risk group defined by the median of normalized read counts across the patients comprised most of patients with FL in the study. Kaplan-Meier survival curves are shown for (**a**) CNN2; (**b**) ACRBP; (**c**) IZUMO1; (**d**) HMG20; (**e**) Age; (**f**) Gender; and (**g**) Stage (n=57 with data). DLBCL: diffuse large B-cell lymphoma; FL: follicular lymphoma.

Supplementary Table 1. Differentially modified genes between DLBCL and FL

Gene Symbol	Base Mean	Log2 FC	P-value	Empirical P-value
WASH1	400.669	-0.840	1.08E-05	< 0.001
SPDYE3	192.054	-0.819	1.55E-05	< 0.001
SMPD2	24.786	-0.675	1.74E-05	< 0.001
GTF2IRD2	624.614	-0.607	2.92E-05	< 0.001
GTF2IRD2B	649.582	-0.598	3.49E-05	< 0.001
GOLGA8N	134.258	-0.885	5.07E-05	< 0.001
AC005488.1	60.932	-0.682	5.30E-05	0.005
PGA4	100.853	-1.047	6.24E-05	< 0.001
NPIPB11	348.406	-0.866	7.02E-05	0.001
HIST2H4A	68.287	-0.501	7.52E-05	0.001
PNLIPRP1	23.570	0.669	7.95E-05	0.001
GOLGA8Q	108.272	-0.961	1.03E-04	< 0.001
HIC2	402.991	-0.705	1.03E-04	< 0.001
SLC12A1	98.300	0.583	1.05E-04	< 0.001
RP11-347C12.1	585.969	-0.964	1.15E-04	0.001
NUTM2E	137.636	-0.939	1.20E-04	< 0.001
NPIPB5	818.824	-0.827	1.38E-04	0.012
DDX4	59.640	0.503	1.45E-04	< 0.001
RP11-231C14.4	446.883	-0.970	1.69E-04	0.001
AC006014.1	61.575	-0.653	1.85E-04	0.002
ZNF615	20.587	0.729	1.90E-04	< 0.001
FAM27E3	27.563	-0.955	2.35E-04	< 0.001
NPIPB3	744.473	-0.853	2.54E-04	0.003
HYAL4	52.328	0.606	2.63E-04	< 0.001
PGA5	91.385	-0.746	2.73E-04	< 0.001
PGA3	100.985	-0.964	2.78E-04	< 0.001
PDPK1	735.747	-0.441	3.38E-04	< 0.001
TMEM67	110.781	0.422	3.41E-04	< 0.001
BVES	55.909	0.442	3.41E-04	< 0.001
NOMO3	508.332	-0.569	3.42E-04	0.007
NOMO2	508.116	-0.611	3.42E-04	0.014
AGGF1	136.261	-0.469	3.61E-04	0.003
GOLGA8R	107.913	-0.878	4.45E-04	< 0.001
SPDYE6	135.939	-0.837	5.04E-04	0.001
CDC20	33.641	-0.480	5.12E-04	0.002
EPHX4	39.602	0.609	5.14E-04	< 0.001

SPDYE1	96.909	-0.693	5.38E-04	0.002
NUTM2A	181.884	-0.876	5.42E-04	< 0.001
NUTM2D	189.728	-0.829	5.50E-04	< 0.001
RP13-996F3.5	40.795	-0.875	5.65E-04	0.006
DCN	28.380	0.694	6.05E-04	< 0.001
RIMBP3C	39.078	-0.883	6.27E-04	< 0.001
GRAPL	320.449	-0.662	6.58E-04	< 0.001
IZUMO1	56.478	-0.452	6.59E-04	< 0.001
C1orf185	73.397	0.453	6.80E-04	< 0.001
NPIPB4	731.861	-0.809	6.84E-04	0.008
RP11-449H3.3	72.305	0.411	6.96E-04	< 0.001
KDM4D	24.503	0.654	6.99E-04	< 0.001
LGALS9B	127.701	-0.763	7.02E-04	< 0.001
ZNF30	36.181	0.466	7.03E-04	< 0.001
CCDC172	32.649	0.691	7.07E-04	< 0.001
FRZB	41.535	0.650	7.07E-04	< 0.001
NPIPB6	256.257	-0.806	7.12E-04	0.033
GATSL2	319.820	-0.650	7.16E-04	< 0.001
RIMBP3B	47.212	-0.929	8.26E-04	< 0.001
RP11-162P23.2	76.165	-0.397	8.54E-04	< 0.001
GABRB3	231.695	0.748	8.74E-04	< 0.001
NETO1	72.810	0.682	9.29E-04	< 0.001
STATH	3.239	1.373	9.38E-04	< 0.001
TAS2R1	49.131	0.750	1.02E-03	< 0.001
GRAP	275.418	-0.605	1.03E-03	< 0.001
SLC29A1	120.700	-0.433	1.03E-03	< 0.001
ELMOD1	81.808	0.561	1.04E-03	< 0.001
NPIPB15	200.244	-0.859	1.05E-03	0.017
GPR137C	95.325	0.499	1.05E-03	< 0.001
GOLGA8O	114.201	-0.791	1.08E-03	< 0.001
MARK1	112.462	0.597	1.10E-03	< 0.001
TMEM63A	327.635	-0.349	1.10E-03	< 0.001
GRK5	1961.746	-0.343	1.11E-03	< 0.001
SCNN1G	54.597	0.448	1.12E-03	0.001
USP45	165.113	0.285	1.16E-03	< 0.001
SLC25A21	319.943	0.702	1.16E-03	< 0.001
FBXW10	194.692	-0.360	1.17E-03	0.002
C17orf97	187.263	-0.410	1.20E-03	< 0.001
COL9A1	57.853	0.641	1.21E-03	< 0.001

CUL4A	383.531	-0.412	1.23E-03	< 0.001
GOLGA8I	71.306	-0.582	1.25E-03	0.037
ABCA12	125.089	0.691	1.29E-03	< 0.001
CROCC	1415.484	-0.350	1.30E-03	< 0.001
MUC1	79.686	-1.139	1.32E-03	< 0.001
RNASE10	54.349	-0.727	1.32E-03	< 0.001
WDR6	85.307	-0.417	1.34E-03	< 0.001
GATSL1	321.535	-0.596	1.36E-03	< 0.001
SYT10	36.040	0.804	1.38E-03	< 0.001
CYB5D2	225.161	-0.377	1.38E-03	< 0.001
SPRR3	1.845	1.665	1.39E-03	< 0.001
KRT25	8.029	0.730	1.40E-03	< 0.001
NMS	20.299	0.618	1.41E-03	0.001
SLX1B	65.901	-0.748	1.44E-03	< 0.001
ANKRD11	3289.783	-0.353	1.47E-03	< 0.001
NUTM2B	182.161	-0.863	1.48E-03	< 0.001
TRIM73	158.722	-0.574	1.52E-03	< 0.001
ELAVL2	79.521	0.848	1.53E-03	< 0.001
C10orf90	319.901	0.396	1.54E-03	< 0.001
CDCA4	124.237	-0.372	1.57E-03	< 0.001
VAT1L	274.231	0.406	1.57E-03	< 0.001
RP11-1212A22.4	361.916	-0.983	1.62E-03	0.003
FAT3	435.831	0.572	1.64E-03	< 0.001
CDH20	225.865	0.435	1.66E-03	< 0.001
NPHP1	122.455	0.349	1.69E-03	< 0.001
ZNF471	14.080	0.867	1.71E-03	< 0.001
ANGPTL5	22.495	0.589	1.72E-03	< 0.001
TRIM74	96.171	-0.542	1.73E-03	< 0.001
ATRNL1	529.186	0.637	1.77E-03	< 0.001
COL8A1	147.486	0.565	1.80E-03	< 0.001
ZNF549	13.186	0.597	1.81E-03	0.026
LRRC66	28.860	0.616	1.86E-03	< 0.001
NPIPA1	1494.476	-0.750	1.87E-03	< 0.001
CDRT15L2	9.696	-0.830	1.89E-03	0.002
GOPC	60.444	0.470	1.89E-03	< 0.001
RIMBP3	59.058	-0.730	1.90E-03	< 0.001
OLIG1	3.708	1.074	1.90E-03	0.012
SPDYE5	109.140	-0.642	1.91E-03	0.002
CLEC3A	53.941	0.438	1.92E-03	< 0.001

SLC22A2	129.244	0.517	1.93E-03	< 0.001
PPP2R3A	313.521	0.315	1.96E-03	< 0.001
MUC4	597.371	-0.526	1.96E-03	< 0.001
HOMER1	169.971	0.407	2.02E-03	< 0.001
AASS	82.851	0.676	2.02E-03	< 0.001
BBS7	55.513	0.415	2.02E-03	< 0.001
SHTN1	332.318	0.447	2.04E-03	< 0.001
IQUB	78.531	0.478	2.04E-03	< 0.001
C4A	145.407	-0.771	2.08E-03	< 0.001
GABRB2	142.447	0.677	2.12E-03	< 0.001
GRM8	547.466	0.654	2.12E-03	< 0.001
KCNAB3	32.617	-0.470	2.19E-03	< 0.001
TRIM64C	2.636	1.424	2.21E-03	< 0.001
SHCBP1L	67.351	0.452	2.23E-03	< 0.001
PTPRR	250.291	0.539	2.23E-03	< 0.001
TBC1D3	305.261	-0.760	2.24E-03	0.001
SPDYE16	87.518	-0.627	2.25E-03	0.011
AC138969.4	726.655	-0.979	2.27E-03	< 0.001
ACRBP	80.754	-0.410	2.29E-03	< 0.001
CACNA2D1	363.328	0.561	2.31E-03	< 0.001
EDIL3	310.866	0.545	2.35E-03	< 0.001
PLPP4	139.822	0.434	2.35E-03	0.003
PAK7	266.000	0.557	2.35E-03	< 0.001
RHCE	426.348	-0.404	2.42E-03	0.002
SULT1A3	78.193	-0.645	2.43E-03	< 0.001
CDH19	62.540	0.658	2.46E-03	< 0.001
HIST2H4B	67.979	-0.359	2.46E-03	0.002
RELN	386.353	0.620	2.47E-03	< 0.001
UQCRH	108.239	-0.338	2.47E-03	< 0.001
HMG20B	114.889	-0.505	2.47E-03	< 0.001
PRKD1	474.335	0.564	2.48E-03	< 0.001
KCND2	262.606	0.703	2.48E-03	< 0.001
SYCP1	95.538	0.559	2.48E-03	< 0.001
BOLA2B	4.295	-1.182	2.49E-03	0.048
ABCC2	305.558	-0.373	2.54E-03	0.012
WFDC8	27.169	0.593	2.54E-03	< 0.001
RP13-996F3.4	34.954	-0.780	2.55E-03	0.001
EYA1	106.905	0.714	2.56E-03	< 0.001
IGFL2	18.423	0.591	2.59E-03	< 0.001

DPH7	198.568	-0.647	2.61E-03	< 0.001
PGR	55.798	0.757	2.65E-03	< 0.001
BET1L	326.056	-0.387	2.65E-03	< 0.001
KCNIP4	781.537	0.610	2.68E-03	< 0.001
ANKRD33B	938.116	-0.357	2.70E-03	< 0.001
ADCY8	183.573	0.654	2.72E-03	< 0.001
CTNNA2	939.942	0.687	2.73E-03	< 0.001
AL162389.1	15.452	-1.149	2.78E-03	< 0.001
TSPAN8	272.874	0.499	2.81E-03	< 0.001
KCNJ3	128.722	0.703	2.84E-03	< 0.001
ADAMTS19	208.362	0.542	2.85E-03	< 0.001
DPP10	782.868	0.742	2.88E-03	< 0.001
ACOT2	70.009	-0.446	2.89E-03	< 0.001
GOLGA8H	76.666	-0.681	2.89E-03	0.002
ALKBH1	74.081	0.356	2.91E-03	< 0.001
POPDC3	24.212	0.545	2.91E-03	< 0.001
TENM3	620.918	0.470	2.92E-03	< 0.001
PAPOLG	103.587	0.288	2.93E-03	< 0.001
CNN2	222.520	-0.560	2.94E-03	< 0.001
NKAIN3	476.361	0.652	2.94E-03	< 0.001
NRG3	642.218	0.708	2.96E-03	< 0.001
TMEM132C	448.234	0.490	2.98E-03	0.005
GOLGA6L22	66.072	-0.699	3.00E-03	0.001
CEP126	77.258	0.501	3.01E-03	< 0.001
STK16	48.521	-0.486	3.01E-03	< 0.001
SLC34A1	137.031	-0.364	3.01E-03	< 0.001
VWC2L	103.907	0.690	3.03E-03	< 0.001
RUNDC3B	127.141	0.460	3.08E-03	< 0.001
SOSTDC1	97.777	0.410	3.11E-03	< 0.001
SLC35F1	289.553	0.601	3.13E-03	< 0.001
CCDC59	85.478	0.552	3.13E-03	< 0.001
ASZ1	47.639	0.571	3.15E-03	< 0.001
GOLGA8J	75.719	-0.706	3.15E-03	0.003
ZNF226	18.571	0.515	3.15E-03	0.001
PCDH7	242.682	0.659	3.18E-03	< 0.001
FREM2	149.758	0.589	3.20E-03	< 0.001
GLT6D1	16.389	0.692	3.21E-03	< 0.001
PACRGL	41.524	0.538	3.22E-03	< 0.001
HAPLN1	56.851	0.593	3.22E-03	< 0.001

SLX1A	67.911	-0.663	3.24E-03	< 0.001
GABRG2	35.752	0.885	3.24E-03	< 0.001
AL591479.1	35.233	-0.704	3.25E-03	< 0.001
CSMD3	549.749	0.675	3.25E-03	< 0.001
LASP1	615.540	-0.352	3.26E-03	< 0.001
LRRIQ1	110.972	0.675	3.26E-03	< 0.001
SH3GL2	156.546	0.745	3.29E-03	< 0.001
SLIT2	258.397	0.547	3.29E-03	< 0.001
TCTN3	54.569	0.408	3.30E-03	< 0.001
CFAP70	154.657	0.351	3.31E-03	< 0.001
SIRPA	413.505	-0.363	3.34E-03	< 0.001
TMEM173	68.143	-0.355	3.37E-03	< 0.001
ADAD1	37.282	0.631	3.39E-03	< 0.001
LUZP2	357.354	0.587	3.39E-03	< 0.001
TYR	48.752	0.927	3.40E-03	< 0.001
THSD7B	529.490	0.763	3.42E-03	< 0.001
GOLGA8T	72.001	-0.772	3.43E-03	0.003
RIT2	201.027	0.678	3.43E-03	< 0.001
ANKS1B	837.193	0.615	3.44E-03	< 0.001
NYAP2	191.172	0.534	3.44E-03	< 0.001
ANO4	270.523	0.651	3.45E-03	< 0.001
PTPRD	1898.105	0.496	3.48E-03	< 0.001
AL627309.1	152.715	-0.975	3.50E-03	< 0.001
LCE2B	1.542	1.594	3.52E-03	0.001
GRM1	337.403	0.538	3.54E-03	< 0.001
NPSR1	165.781	0.539	3.55E-03	< 0.001
VTCN1	81.268	0.408	3.55E-03	< 0.001
GJB2	46.991	-0.517	3.57E-03	< 0.001
TRPC4	195.635	0.570	3.57E-03	< 0.001
SLC35F4	263.491	0.704	3.60E-03	< 0.001
ADAMTS18	151.496	0.599	3.62E-03	< 0.001
PCDH15	926.690	0.647	3.66E-03	< 0.001
OR4K1	0.940	1.848	3.66E-03	< 0.001
LRP1B	1049.304	0.633	3.67E-03	< 0.001
NELL1	541.508	0.743	3.72E-03	< 0.001
CCDC39	166.213	0.625	3.81E-03	< 0.001
GRM7	972.404	0.480	3.81E-03	< 0.001
NLRP8	44.182	0.788	3.84E-03	0.008
FAM234A	133.330	-0.328	3.84E-03	< 0.001

GUCY1A2	170.001	0.738	3.86E-03	< 0.001
PAX3	76.498	0.528	3.92E-03	< 0.001
NPIPA8	549.397	-0.921	3.92E-03	< 0.001
EPHA1	192.456	-0.431	3.93E-03	< 0.001
CA3	98.254	0.441	3.93E-03	< 0.001
HSPB8	72.583	0.392	3.94E-03	0.002
ADAM7	91.653	0.466	3.95E-03	< 0.001
NMUR2	20.871	0.854	3.96E-03	< 0.001
GLRB	90.292	0.508	3.96E-03	< 0.001
DYNC1I1	426.744	0.388	3.96E-03	< 0.001
SGCZ	722.613	0.552	4.00E-03	< 0.001
TTC29	146.273	0.631	4.00E-03	< 0.001
DOK5	194.850	0.391	4.00E-03	< 0.001
RIMS1	278.914	0.674	4.02E-03	< 0.001
SLC43A3	33.770	-0.451	4.04E-03	< 0.001
ZNF396	23.309	0.399	4.06E-03	< 0.001
CA10	359.111	0.661	4.07E-03	< 0.001
UMOD	31.712	0.537	4.11E-03	0.048
BRMS1L	200.100	0.282	4.14E-03	< 0.001
WDR19	197.248	0.311	4.17E-03	< 0.001
NLGN1	749.363	0.504	4.18E-03	< 0.001
NCF1	216.557	-0.550	4.20E-03	< 0.001
RCC1	304.556	-0.334	4.21E-03	< 0.001
CDC42SE1	65.002	-0.325	4.22E-03	< 0.001
TMPRSS11E	56.765	0.543	4.25E-03	< 0.001
TMC6	301.423	-0.435	4.25E-03	< 0.001
NUDT1	134.161	-0.518	4.27E-03	< 0.001
RBFOX1	1340.282	0.595	4.33E-03	< 0.001
PDE1A	285.494	0.555	4.34E-03	< 0.001
TMPRSS15	151.497	0.560	4.34E-03	< 0.001
UNC13C	435.392	0.598	4.36E-03	< 0.001
KCTD8	129.121	0.768	4.38E-03	< 0.001
CADPS	355.599	0.595	4.38E-03	< 0.001
ZIC1	64.747	0.625	4.39E-03	< 0.001
GRM5	393.271	0.566	4.41E-03	< 0.001
BMP5	50.401	0.815	4.44E-03	< 0.001
PTPN21	150.235	0.341	4.47E-03	< 0.001
RYR2	970.424	0.326	4.51E-03	< 0.001
PPP1R9A	359.634	0.434	4.53E-03	< 0.001

CNTN4	704.233	0.575	4.53E-03	< 0.001
YAE1D1	111.936	0.331	4.55E-03	< 0.001
PPIB	47.788	-0.428	4.56E-03	< 0.001
FAM19A1	291.394	0.720	4.62E-03	< 0.001
C17orf62	253.742	-0.721	4.64E-03	< 0.001
KIAA1143	29.958	0.565	4.70E-03	< 0.001
GOLGA6L6	57.488	-0.771	4.70E-03	0.001
RP11-467N20.5	78.988	-0.776	4.70E-03	< 0.001
AQP12B	81.467	-0.726	4.78E-03	< 0.001
CNTN6	202.903	0.545	4.80E-03	< 0.001
ROBO2	1046.077	0.642	4.81E-03	< 0.001
UNC5D	361.962	0.626	4.85E-03	< 0.001
SLC17A6	20.089	0.769	4.85E-03	0.001
MYH9	1677.690	-0.368	4.85E-03	< 0.001
ZNF248	87.797	0.472	4.87E-03	< 0.001
ARHGEF10	1099.326	-0.331	4.88E-03	< 0.001
GPM6A	223.462	0.613	4.88E-03	< 0.001
CCBE1	276.226	0.511	4.91E-03	< 0.001
TBC1D3G	356.665	-0.709	4.91E-03	0.012
ABHD15	48.887	-0.383	4.93E-03	< 0.001
DEFB125	8.818	0.736	4.93E-03	< 0.001
IL21	16.045	0.614	4.97E-03	< 0.001
HS3ST4	338.669	0.626	4.97E-03	< 0.001
SULT1A4	77.607	-0.617	4.98E-03	< 0.001

Notes:
FC: Fold Change. Fold change > 0 refers to DLBCL up-modified genes; fold change <0 refers to FL up-modified genes.

Supplementary Table 2. Cytobands enriched by differentially modified genes between DLBCL and FL

Cytoband	Count	P-value	Genes	FDR %
15q13.2	6	5.788E-07	GOLGA8T, GOLGA8H, GOLGA8J, GOLGA8Q, GOLGA8O, GOLGA8N	0.001
7q11.23	9	2.009E-06	SPDYE1, GATSL2, NCF1, GTF2IRD2, SPDYE5, SPDYE6, GTF2IRD2B, TRIM74, TRIM73	0.003
15q13.3	5	9.321E-05	GOLGA8H, GOLGA8J, GOLGA8Q, GOLGA8O, GOLGA8N	0.119
16p11.2	8	8.333E-04	HS3ST4, SLX1B, SULT1A3, BOLA2B, SLX1A, SULT1A4, NPIPB11, NPIPB6	1.062

Supplementary Table 3. Linking local regulatory elements with four signature genes from 5hmC profiling

Gene Label	Position - Gene Body	Genomic Region - H3K4me1	r	P-value
**ACRBP**	chr12: 6,747,241-6,756,626	chr12: 6,744,061-6,756,545	0.593	3.32E-08
**IZUMO1**	chr19: 49,244,109-49,250,166	chr19: 49,244,356-49,250,875	0.506	4.81E-06
**CNN2**	chr19: 1,026,298-1,039,068	chr19: 1,025,867-1,039,955	0.650	2.83E-10
**HMG20B**	chr19: 3,572,775-3,579,086	chr19: 3,573,325-3,576,566	0.688	1.73E-11

Gene Label	Position - Gene Body	Genomic Region - H3K27ac	r	P-value
**ACRBP**	chr12: 6,747,241-6,756,626	chr12: 6,747,151-6,756,549	0.417	2.40E-04
**IZUMO1**	chr19: 49,244,109-49,250,166	NA		
**CNN2**	chr19: 1,026,298-1,039,068	chr19: 1,026,067-1,039,844	0.624	3.90E-09
**HMG20B**	chr19: 3,572,775-3,579,086	chr19: 3,572,933-3,573,192	0.177	0.13

Gene Label	Position - Gene Body	Genomic Region - H3K9me3	r	P-value
**ACRBP**	chr12: 6,747,241-6,756,626	NA		
**IZUMO1**	chr19: 49,244,109-49,250,166	chr19: 49,244,785-49,250,346	0.594	2.98E-08
**CNN2**	chr19: 1,026,298-1,039,068	NA		
**HMG20B**	chr19: 3,572,775-3,579,086	NA		

Gene Label	Position - Gene Body	Genomic Region - H3K27me3	r	P-value
**ACRBP**	chr12: 6,747,241-6,756,626	NA		
**IZUMO1**	chr19: 49,244,109-49,250,166	chr19: 49,244,025-49,250,845	0.218	0.06
**CNN2**	chr19: 1,026,298-1,039,068	NA		
**HMG20B**	chr19: 3,572,775-3,579,086	NA		

Notes:
Chromosome positions of gene body and histone modifications are based on hg19 reference

Supplementary Table 4. Enriched KEGG pathways and GO biological processes

Term	Count	P-value	Genes	FDR
**173 differentially modified genes with higher 5hmC in DLBCL relative to FL**				
KEGG:hsa04723:Retrograde endocannabinoid signaling	9	5.27E-07	GABRB3, GABRB2, RIMS1, GRM5, SLC17A6, ADCY8, GABRG2, GRM1, KCNJ3	4.37E-05
KEGG:hsa04724:Glutamatergic synapse	8	1.58E-05	GRM5, HOMER1, GRM7, GRM8, SLC17A6, ADCY8, GRM1, KCNJ3	6.57E-04
KEGG:hsa05032:Morphine addiction	6	4.81E-04	GABRB3, GABRB2, PDE1A, ADCY8, GABRG2, KCNJ3	1.33E-02
KEGG:hsa04080:Neuroactive ligand-receptor interaction	9	7.69E-04	GABRB3, GABRB2, GRM5, GRM7, GLRB, GRM8, NMUR2, GABRG2, GRM1	1.60E-02
GO:0007268~Chemical synaptic transmission	11	6.72E-05	PTPRD, GABRB2, UNC13C, GRM5, RIT2, HOMER1, GRM7, KCND2, GLRB, SYT10, GRM1	4.57E-02
GO:0007389~Pattern specification process	5	1.12E-04	EYA1, NRG3, ZIC1, SOSTDC1, BMP5	4.57E-02
**121 differentially modified genes with higher 5hmC in FL relative to DLBCL**				
GO:0044267~cellular protein metabolic process	6	1.24E-04	HIST2H4A, HIST2H4B, SLC34A1, PGA3, PGA5, PGA4	4.68E-02

Notes:
KEGG: Kyoto Encyclopedia of Genes and Genomes; GO: Gene Ontology; FDR: False Discovery Rate
