# Supplementary material for: Alterations of 5-hydroxymethylation in circulating cell-free DNA reflect molecular distinctions of subtypes of non-Hodgkin lymphoma
Source: NPJ Genom Med. 2021 Feb 11;6:11. doi: 10.1038/s41525-021-00179-8 (PMC7878492; doi:10.1038/s41525-021-00179-8)
Supplement: Supplementary file 2 — Reporting Summary [file 41525_2021_179_MOESM2_ESM.pdf]

nature research

Corresponding author(s): DBPR NPJGENMED-00683R

Last updated by author(s): Jan 7, 2021

Reporting Summary

Nature Research wishes to improve the reproducibility of the work that we publish. This form provides structure for consistency and transparency in reporting. For further information on Nature Research policies, see our Editorial Policies and the Editorial Policy Checklist.

Statistics

For all statistical analyses, confirm that the following items are present in the figure legend, table legend, main text, or Methods section.

n/a	Confirmed	
☐	☒	The exact sample size (*n*) for each experimental group/condition, given as a discrete number and unit of measurement
☒	☐	A statement on whether measurements were taken from distinct samples or whether the same sample was measured repeatedly
☐	☒	The statistical test(s) used AND whether they are one- or two-sided<br>*Only common tests should be described solely by name; describe more complex techniques in the Methods section.*
☐	☒	A description of all covariates tested
☐	☒	A description of any assumptions or corrections, such as tests of normality and adjustment for multiple comparisons
☐	☒	A full description of the statistical parameters including central tendency (e.g. means) or other basic estimates (e.g. regression coefficient) AND variation (e.g. standard deviation) or associated estimates of uncertainty (e.g. confidence intervals)
☐	☒	For null hypothesis testing, the test statistic (e.g. *F*, *t*, *r*) with confidence intervals, effect sizes, degrees of freedom and *P* value noted<br>*Give P values as exact values whenever suitable.*
☒	☐	For Bayesian analysis, information on the choice of priors and Markov chain Monte Carlo settings
☒	☐	For hierarchical and complex designs, identification of the appropriate level for tests and full reporting of outcomes
☒	☐	Estimates of effect sizes (e.g. Cohen's *d*, Pearson's *r*), indicating how they were calculated

Our web collection on statistics for biologists contains articles on many of the points above.

Software and code

Policy information about availability of computer code

Data collection	*Provide a description of all commercial, open source and custom code used to collect the data in this study, specifying the version used OR state that no software was used.*
Data analysis	R Statistical Environment (open source)

For manuscripts utilizing custom algorithms or software that are central to the research but not yet described in published literature, software must be made available to editors and reviewers. We strongly encourage code deposition in a community repository (e.g. GitHub). See the Nature Research guidelines for submitting code & software for further information.

Data

Policy information about availability of data

All manuscripts must include a data availability statement. This statement should provide the following information, where applicable:

- Accession codes, unique identifiers, or web links for publicly available datasets
- A list of figures that have associated raw data
- A description of any restrictions on data availability

The individual-level raw and processed 5hmC-Seal profiles have been deposited into the NCBI Gene Expression Omnibus database (Accession Number: GSE155228).

Field-specific reporting

Please select the one below that is the best fit for your research. If you are not sure, read the appropriate sections before making your selection.

☒ Life sciences ☐ Behavioural & social sciences ☐ Ecological, evolutionary & environmental sciences

For a reference copy of the document with all sections, see nature.com/documents/nr-reporting-summary-flat.pdf

Life sciences study design

All studies must disclose on these points even when the disclosure is negative.

Sample size	We prospectively enrolled adult patients ≥20 years old who were newly diagnosed with non-Hodgkin lymphoma (NHL) at the University of Chicago Medical Center (UCMC) from 2010 to 2013. Blood samples were drawn from consented patients. A total of 73 patients (de novo DLBCL, n = 48; FL, n = 25) with plasma available for cfDNA extraction was included in this study.
Data exclusions	Patients with primary central nervous system lymphoma, post-transplant lymphoproliferative disorder, transformation of a previously diagnosed indolent lymphoma, or HIV infection were excluded.
Replication	n/a
Randomization	n/a (this study is an epidemiology observational study)
Blinding	n/a

Reporting for specific materials, systems and methods

We require information from authors about some types of materials, experimental systems and methods used in many studies. Here, indicate whether each material, system or method listed is relevant to your study. If you are not sure if a list item applies to your research, read the appropriate section before selecting a response.

Materials & experimental systems

n/a	Involved in the study	
☒	☐	Antibodies
☒	☐	Eukaryotic cell lines
☒	☐	Palaeontology and archaeology
☒	☐	Animals and other organisms
☐	☒	Human research participants
☐	☒	Clinical data
☒	☐	Dual use research of concern

Methods

n/a	Involved in the study	
☒	☐	ChIP-seq
☒	☐	Flow cytometry
☒	☐	MRI-based neuroimaging

Human research participants

Policy information about studies involving human research participants

Population characteristics	The current report included 73 patients (de novo diffuse large B-cell lymphoma [DLBCL] = 48; follicular lymphoma [FL] = 25) with plasma available for cell-free DNA extraction. There were no significant differences between DLBCL (n = 48) and FL (n = 25) with respect to gender distribution, race/ethnicity, and stage. The median age at the time of diagnosis for patients with FL (51.0 yrs) was lower than that of DLBCL (59.0 yrs). Among the 48 patients with DLBCL, 34 had cell-of-origin determined based on the Han's algorithm. Of those, 23 were germinal center B-cell-like (GCB) DLBCL and 11 patients were activated B-cell-like (ABC) DLBCL. In addition, 28 (38.4%) had an elevated LDH levels (cut-off: ≥ 245U/L) at the time of diagnosis.
Recruitment	We prospectively enrolled adult patients ≥20 years old who were newly diagnosed with non-Hodgkin lymphoma (NHL) at the University of Chicago Medical Center (UCMC) from 2010 to 2013. All diagnoses were confirmed by hematopathologists according to the 2008 World Health Organization criteria. Blood samples were drawn from consented patients and processed immediately to separate plasma. Patients with primary central nervous system lymphoma, post-transplant lymphoproliferative disorder, transformation of a previously diagnosed indolent lymphoma, or HIV infection were excluded.
Ethics oversight	This study was approved by the Institutional Review Board of the University of Chicago.

Note that full information on the approval of the study protocol must also be provided in the manuscript.

Clinical data

Policy information about clinical studies

All manuscripts should comply with the ICMJE guidelines for publication of clinical research and a completed CONSORT checklist must be included with all submissions.

Clinical trial registration	This study is not a clinical trial. It is an epidemiology observational study. Clinical data were obtained from electronic health records.
Study protocol	N/A
Data collection	Data on clinical and pathologic characteristics of subjects at the time of diagnosis, such as lactic acid dehydrogenase (LDH) level, Ann Arbor stage, and tumor cell-of-origin were collected from electronic health records.
Outcomes	Deaths were determined using the National Death Index. Overall survival was defined as the time from initial diagnosis until death from any cause. Follow-up was through December 31, 2017.
